# Design of an Artificial Neural Network Algorithm for a Low-Cost Insole Sensor to Estimate the Ground Reaction Force (GRF) and Calibrate the Center of Pressure (CoP)

**DOI:** 10.3390/s18124349

**Published:** 2018-12-10

**Authors:** Ho Seon Choi, Chang Hee Lee, Myounghoon Shim, Jong In Han, Yoon Su Baek

**Affiliations:** Motion Control Laboratory, School of Mechanical Engineering, Yonsei University, Seoul 03722, Korea; eggr@yonsei.ac.kr (H.S.C.); c15606@yonsei.ac.kr (C.H.L.); smh2119@yonsei.ac.kr (M.S.); gmiilp1318@naver.com (J.I.H.)

**Keywords:** center of pressure (CoP), force sensing resistor (FSR), ground reaction force (GRF), artificial neural network (ANN)

## Abstract

As an alternative to high-cost shoe insole pressure sensors that measure the insole pressure distribution and calculate the center of pressure (CoP), researchers developed a foot sensor with FSR sensors on the bottom of the insole. However, the calculations for the center of pressure and ground reaction force (GRF) were not sufficiently accurate because of the fundamental limitations, fixed coordinates and narrow sensing areas, which cannot cover the whole insole. To address these issues, in this paper, we describe an algorithm of virtual forces and corresponding coordinates with an artificial neural network (ANN) for low-cost flexible insole pressure measurement sensors. The proposed algorithm estimates the magnitude of the GRF and the location of the foot plantar CoP. To compose the algorithm, we divided the insole area into six areas and created six virtual forces and the corresponding coordinates. We used the ANN algorithm with the input of magnitudes of FSR sensors, 1st and 2nd derivatives of them to estimate the virtual forces and coordinates. Eight healthy males were selected for data acquisition. They performed an experiment composed of the following motions: standing with weight shifting, walking with 1 km/h and 2 km/h, squatting and getting up from a sitting position to a standing position. The ANN for estimating virtual forces and corresponding coordinates was fitted according to those data, converted to c script, and downloaded to a microcontroller for validation experiments in real time. The results showed an average RMSE the whole experiment of 31.154 N for GRF estimation and 8.07 mm for CoP calibration. The correlation coefficients of the algorithm were 0.94 for GRF, 0.92 and 0.76 for the X and Y coordinate respectively.

## 1. Introduction

Human postural control is used for maintaining a subject’s balance against the external force of gravity during locomotion [1,2]. By analyzing the sensory information and calculating the biomechanics for the coordinates of limb segments, we can determine the velocity of the center of mass of the segments or the joint torque that can be used for control. These methods are also used in rehabilitation exoskeletons and muscular power strengthening exoskeletons. We should know the state of the wearer for calculating the magnitude and direction of the assistive force for the wearers’ needs.

Foot plantar center of pressure (CoP) and ground reaction force (GRF) provide the most important information for postural control. Perry et al. showed that gravity, which is the only external force during human locomotion, creates the GRF on the insole, which is the end effector of the lower limbs, and contains important information like the state of the human, intention understanding [3]. We can predict the stage of the gait cycle by the change of vertical GRF [4]. It has two peak values: the first one is the maximal weight acceptance and second is the push-off. It starts with the heel contact and ends with the toe-off. The valley between the two peaks is in the mid stance. We can also calculate the velocity of the body mass from a three-dimensional center of mass acceleration, which is determined from the GRF by the center of mass mechanics [5]. After disassembling the GRF, we can get the magnitude of the horizontal force and can calculate the horizontal acceleration with dividing the horizontal force with the body mass. It can also be used for analyzing patients with abnormal gait patterns [6]. During the stance phase, we can judge the person’s gait normality from the trajectory of the x and y coordinates of the center of GRF and for controlling the exoskeletons which are used by those patients [7]. For example, Shaulian et al. proved that the GRF of patients with lower-limb diseases deviates substantially from the norm [8]. So, they concluded that GRF manipulation is a key parameter for treating the symptoms of those diseases. And Ma and Liao modeled and evaluated patients’ gait patterns based on GRF using a semi-Markov process [9]. Lim et al. used an FSR foot sensor for detecting the gait phase algorithm for controlling a lower extremity exoskeleton robot [10].

There are various methods for measuring the magnitude of the GRF. We can use the force plate and in-shoe measurement and the analysis system (e.g., the F-Scan system of TEKSCAN Inc., South Boston, MA, USA or the Insole plantar pressure feedback system of Shenzhen XFT Electronics Co., Ltd., Shenzhen, China). However, these systems can be used only in laboratory experiments, which hinders the mobility of the experiments. They are also expensive (approximately $20,000 for the entire system), so they have disadvantages in terms of cost-effectiveness.

The CoP, which is another informational element for postural control, is often used as much as the GRF. We can judge a person’s state for stability with the location and velocity of the CoP [11,12]. We can also predict the gait speed of a person with the forward velocity of the CoP in the midfoot [13] and get the user intention understanding of exoskeleton for controlling a robot system [14,15]. Nevertheless, we should use an in-shoe pressure sensor as for measuring the GRF, so the same problem exists for this case.

To solve the monetary problems, many alternative sensors have been developed. Park et al. made a Force Sensing Resistor (FSR) foot sensor with four FSR sensors on the insole and obtained the data of the force plate and the FSR sensors simultaneously during walking, running and jumping [16]. Moreover, they composed a linear multiple regression formula with four FSR sensor inputs for GRF estimation with the output of magnitude of GRF. However, they did not remove the hysteresis of the FSR sensors. So, the inputs of the algorithm cannot be corrected most of the time. They obtained the force loss during the stance phase of the gait cycle because they cannot estimate the pressure on the arch of the foot, which has no FSR sensors. So, they get the NRMS error of 14.68 during the walking which cannot be ignored. Moreover, the algorithm they developed can only be utilized during those three motions. For that method, we need to set the mode of motion for operation and cannot use the algorithm when we do not know the state of human locomotion. Forner-Cordero et al. estimated the magnitude of the GRF using the pressure value from limited areas of the FSR sensors [17]. However, they used inverse dynamics because they knew the subject was in the stance phase. Thus, the algorithm can be used only when the state of the subject is known, especially during the stance phase.

FSR sensors are also used for low-cost sensors that calculate the CoP. Normally, a weighted mean approach (WMA) is used for calculating the CoP. The values of FSR sensors are divided by the sum of the FSR sensor values and multiplied by their coordinates like a weight constant. Then they are all summed for the CoP calculation [18]. However, this method is not accurate because of the limited areas of the FSR sensors, which do not cover the entire insole area, and the error cannot be compensated for. To solve this issue, He et al. developed an algorithm estimating the foot plantar CoP with nonlinear models. The output value of nonlinear models is the location of the CoP and the input values are the voltages of the FSR sensors. The author fitted the algorithm with data from the F-Scan system for reference during several tasks [19]. A low-cost foot sensor with 12 FSR sensors was validated for the algorithm during the tasks. However, this method was fitted for static tasks like quiet standing or sit to stand, so its utilization is limited and it may not operate well in dynamic tasks. The researchers estimated the location of the CoP directly from the values of FSR sensors (not through the whole pressure value of the insole) to get the errors from that. Thus, it is not a fundamental solution.

We aimed to develop a low-cost flexible insole pressure measurement sensor that estimates the magnitude of the GRF and calibrates the location of the CoP during static and dynamic motions. We used a WMA, but we created the virtual forces and their coordinates for passing the bounds of limited sensing areas. Then an artificial neural network (ANN) system was used to estimate the magnitude and coordinates of the virtual forces. An ANN algorithm has the inputs of the values of the FSR sensors. For fitting the algorithms, the F-Scan system was used for several tasks. After predicting those values, we summed the virtual forces to estimate the magnitude of the GRF and used the WMA to calibrate the CoP. Validation was also done with the F-Scan system during several tasks.

The remainder of this paper is structured as follows: In Section 2 the hardware and our proposed algorithm design for estimating the virtual forces and moving coordinates are presented. And the settings for ANN are also shown in Section 2. In Section 3 experiments for data acquisition are presented. The experimental results are shown in Section 4 and they are discussed in Section 5. Lastly, conclusion is given in Section 6.

## 2. Methods

### 2.1. Hardware Design

**Figure 1:**
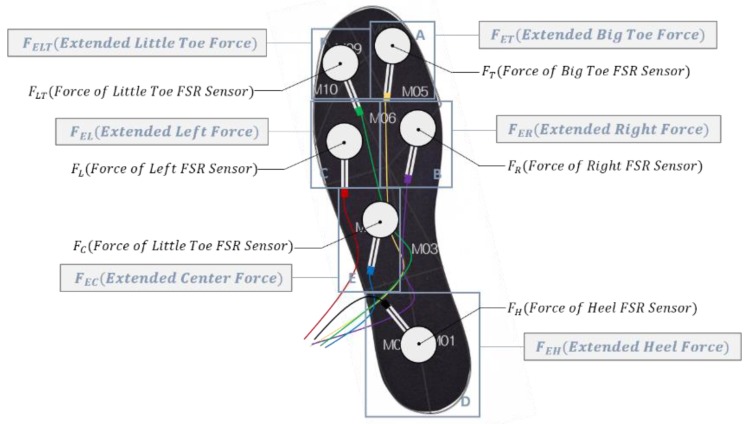
Design of the low-cost flexible insole pressure measurement sensor with six FSR sensors. Gray circles with wires show the FSR sensors. White bolded numbers indicate the anatomically divided areas. Blue squares mean the areas of the virtual forces that contain the FSR sensors in them.

The locations of the FSR sensors are the most important elements for composing the algorithms. Every algorithm needs the values of the FSR sensors as input values, so they should be located appropriately. We set the location of the FSR sensors according to the anatomical shape of a foot, and they are the protruding areas. These areas make contact with the ground earlier than the others and get more pressure than the others, so FSR sensors should be located at these areas for sensing the pressure most of time, and we can utilize these values to estimate the pressure of other areas. We used six FSR sensors, and their locations were at two phalanges, two of metatarsal bones, the calcaneus, and the arch of the foots as shown in Figure 1. The sensor at the arch of the foot(E) was used for another reason. Because pressure at this area is intermittently formed, estimation of it is very difficult without measurements, and because of its shape, this is the last area to get pressure during the locomotion. Therefore, this area for input value was helpful for algorithm fitting. FSR sensors were attached to a polypropylene insole of size US8, and the signals were transmitted to the microcontroller (STM32F407VG-discl1, STMicroelectronics, Geneva, Switzerland) through the circuit, which is recommended by the manufacturer [20].

FSR-A401 (TEKSCAN Inc., South Boston, MA, United States) with a sensing area of 2026.93 ${\mathrm{mm}}^{2}$ was used, and we calibrated them using the system in Hall et al. [21]. After the FSR sensors are used, the electric resistances of the FSR sensors change. Therefore, we fitted the 4th-order polynomial function with the FSR voltage value and, its moving integral, offset to compensate for that and also for reducing the hysteresis effect. To reduce the noise from the sensors, we used an RC analogue high-pass filter with 335.06 Hz cutoff frequency, and on the computer, we also used a digital low-pass filter with 20 Hz cutoff frequency for the noise.

### 2.2. Virtual Force Algorithm (VFA) Design

The insole was divided into six areas to estimate the whole insole pressure. The areas were composed around the FSR sensors, and the FSR sensors were the center of the areas as in Figure 1. Every area had a force converted from the pressure of the area and its moving coordinate, which is shown in Table 1. These values can be predicted from algorithms for which inputs are the values of the FSR sensors. We used an ANN algorithm for the virtual force algorithm (VFA).

The ANN system is a statistical learning algorithm designed from the idea of the nerve network of humans. It is a model that estimates the output values from a node that is composed of numerous synapses and multiplied by fitted weight constants. A human cannot calculate the magnitude of the GRF and the location of the CoP for every moment, but he can maintain the balance of the body and perform locomotion by feeling it with a nerve network that is fitted with numerous frames that accumulated since birth. We can use this point for our algorithms.

**Figure 2:**
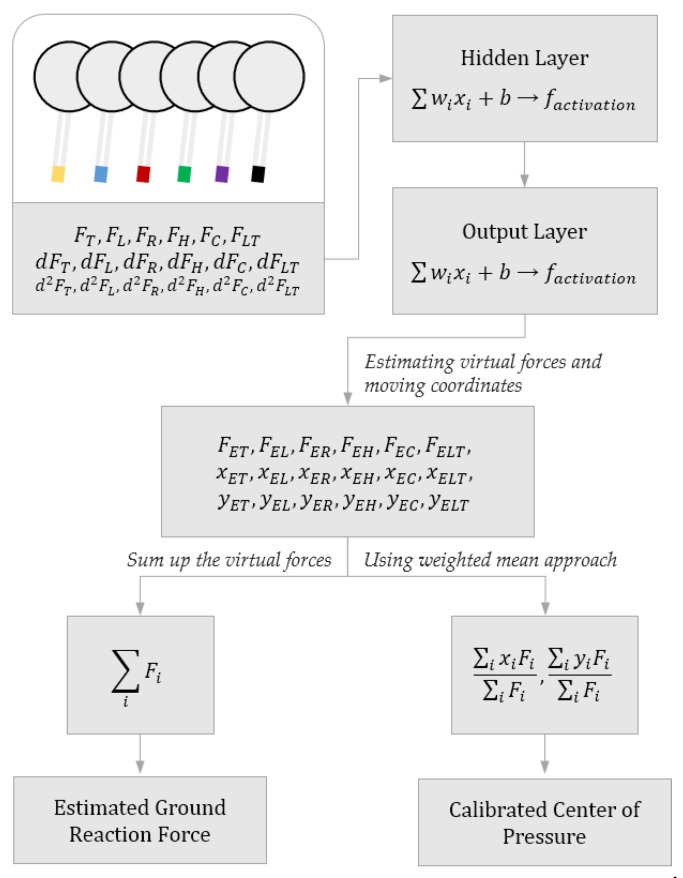
Flow chart of the ANN algorithm for estimating the GRF and calibrating the CoP of the insole.

We trained 18 ANN algorithms with 18 input values of magnitudes, 1st and 2nd derivatives of magnitudes of six FSR sensors for the magnitudes of virtual forces and coordinates of their locations like in Figure 2. We used those values to reduce drastic errors during experiments. They maintain the slope of the estimated values with velocities and accelerations so there can be no abrupt dominant change.

A neural network fitting toolbox in MATLAB (The MathWorks, Inc., Natick, MA, USA) was used for algorithm fitting. Tangent-sigmoid transfer function, which is an activation function for connecting the layers was used for hidden layer and output layer and a Levenberg‒Marquardt backpropagation algorithm was used for training the weighted constants of the algorithm. Collected dataset were divided into three parts: 70% for training, 15% for validation, and 15% for testing.

(1) $$GRF=F_{ET}+F_{EL}+F_{ER}+F_{EH}+F_{EC}+F_{ELT}$$

The magnitudes of the GRF were calculated by summing the virtual forces as in Equation (1), and the location of the foot plantar CoP was calculated by a WMA with virtual forces and their locations as in Equation (2).

(2) $$\begin{array}{l} x_{COP}=\frac{x_{ET}F_{ET}+x_{EL}F_{EL}+x_{ER}F_{ER}+x_{EH}F_{EH}+x_{EC}F_{EC}+x_{ELT}F_{ELT}}{F_{ET}+F_{ER}+F_{EL}+F_{EH}+F_{EC}+F_{ELT}}\\ y_{COP}=\frac{y_{ET}F_{ET}+y_{EL}F_{EL}+y_{ER}F_{ER}+y_{EH}F_{EH}+y_{EC}F_{EC}+y_{ELT}F_{ELT}}{F_{ET}+F_{ER}+F_{EL}+F_{EH}+F_{EC}+F_{ELT}} \end{array}.$$

### 2.3. Artificial Neural Network Setting

**Figure 3:**
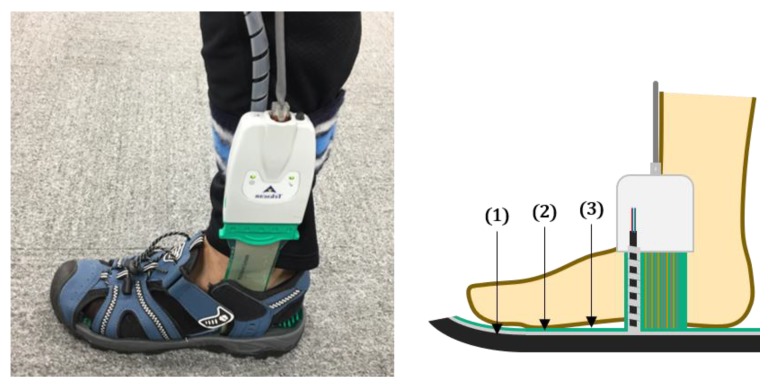
Picture of experimental setup. All experiments were done wearing the F-Scan system in-shoe sensor and low-cost FSR foot sensor simultaneously. (1), (2), (3) represents the shoe, developed FSR foot sensor, and the F-Scan in-shoe sensor respectively. And they are piled up in order.

Before doing the experiments for many subjects, we should decide the number of layers of an ANN. So, we did the scan experiment which is performed standing on two feet and moving the center of mass counter-clockwise and back and forth for one subject. Data for fitting the algorithms were acquired by the F-Scan system. Chen et al. confirmed that the F-Scan system was accurate for measuring GRF and CoP [22]. We used the F-Scan system for reference data. Every experiment was done with the subjects wearing an F-scan insole sensor and low-cost flexible insole pressure measurement sensor as in Hu et al. like Figure 3 [19]. The data acquisition rate was set to 50 Hz (20 ms). After data acquisition, we composed the matrices for algorithm fitting. One frame of 20 ms was converted to one data matrix. The matrix is composed of 19 columns with the six magnitudes of the FSR sensors, six 1st and 2nd derivatives of them for input values, and pressure values and coordinates of the divided. We fitted the ANN algorithm with different number of layers of hidden neurons for finding the optimum number.

**Figure 4:**
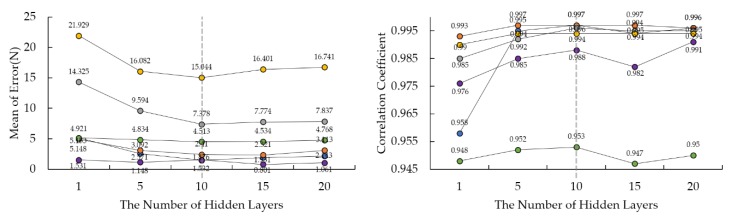
Results of Algorithm Fitting with Different Layers of Hidden Neurons for Virtual Forces. Left graph presents the mean of error resulted by each number of hidden layers and right graph is for correlation coefficient. Each colored circle means each virtual force (Blue-$F_{ET}$, Orange-$F_{ER}$, Grey-$F_{EL}$, Yellow-$F_{EH}$, Purple-$F_{EC}$, Green-$F_{ELT}$ ).

We knew that time durations for all numbers of layers for estimating virtual forces are less than one second and we did not consider the variable for that reason. As to the mean of error and correlation coefficient, 10 layers had the best performance among all numbers of layers as shown in Figure 4. Therefore, we decided the number of layers would be 10 for estimating the virtual forces.

And for the coordinates, we did same process. Time durations were all less than five hundred seconds, so we also did not consider it and got the number of 200 layers as same reason for the virtual force.

**Figure 5:**
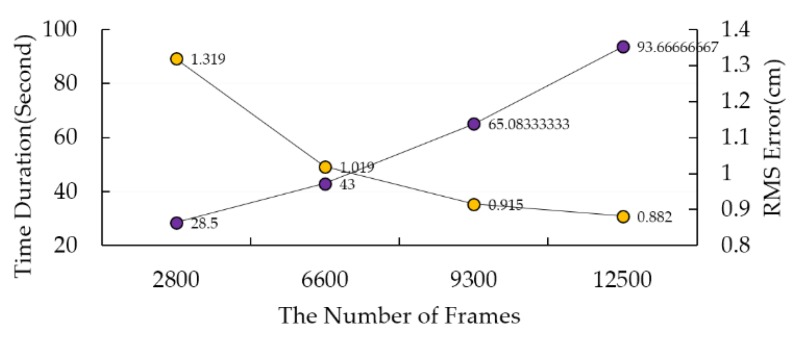
Results of algorithm fitting with different numbers of data frames. Yellow circle means the RMS error and purple one means the time duration of each layer.

We also determined the data amount for algorithm fitting by experiment. Figure 5 shows the result of the algorithm fitting with different numbers of data frames. After 10,000 frames, the rate of error decreasing slowed down. So, we collected 15,000 frames for algorithm fitting and the proportions of each experiment are the same in the data set.

## 3. Experimental Settings

Experiments were done with eight adult males who had a foot size of US8. Their body state indexes are shown in Table 1. Every subject performed five tasks for data acquisition: (1) The scan experiment is performed standing on two feet and moving the center of mass counter-clockwise and back and forth. (2‒3) In the gait cycle experiments the subject walks on a treadmill at 1 km/h and 2 km/h. (4) In the squatting experiment, the subjects squat 10 times. (5) In the Sit to Stand experiment, the subject sat down and got up 10 times.

After fitting the algorithms with acquired data, all algorithms are converted into MATLAB function block of simulink so we can use the algorithm in real time with the values of FSR sensors transmitted from microcontroller (STM32F407VG-disc1, STMicroelectronics, Geneva, Switzerland) board by serial communication (20 ms). We did the whole experimental process again with the algorithm for validation.

## 4. Results

**Figure 6:**
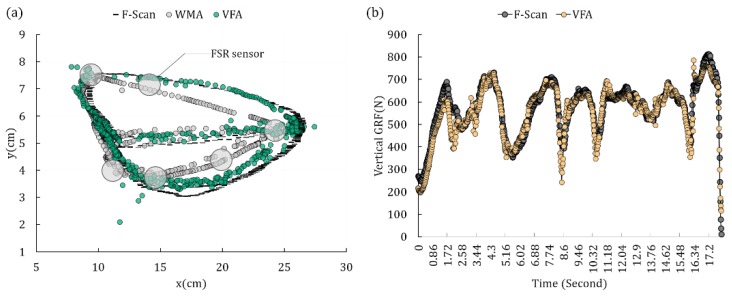
(**a**) The CoP trajectory plot during the scan experiment by the WMA, VFA and F-Scan. The gray circles with bold edges indicate the locations of the FSR sensors. (**b**) One Chosen GRF trajectory plot during the scan experiment by the VFA and F-Scan.

**Figure 7:**
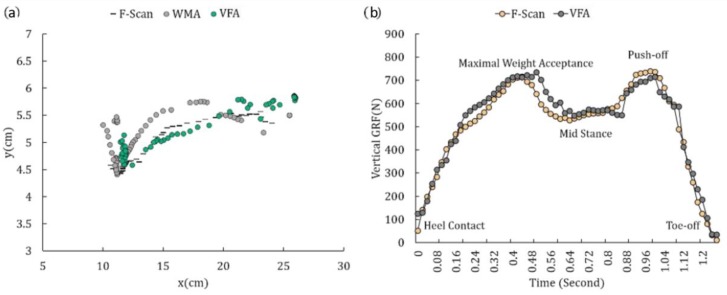
(**a**) One chosen CoP trajectory plot during the stance phase of the 1 km/h gait cycle experiment by the WMA, VFA and F-Scan. (**b**) One chosen GRF trajectory plot during the stance phase of the 1 km/h gait cycle experiment by the VFA and F-Scan.

Figure 6a and Figure 7a show examples of the CoP trajectory plots during the scan experiment, gait experiment of subject 3 by the WMA, the VFA and the reference data of the F-Scan system. We can see that the trajectory of the VFA is much closer to the F-Scan than the WMA. Figure 6b and Figure 7b show the estimation of the magnitude of the GRF during the experiments, and we can see that the estimations are close to the reference data.

Table 2 show the RMSE and correlation coefficients calculated by the VFA and the WMA with the reference data of the F-Scan system. For the GRF, the average RMSE during all the experiments is 29.34 N. There is a value of 8.29 mm for CoP calibration by the VFA and 16.89 mm by the WMA. The average correlation coefficients are 0.94 for the GRF estimation, 0.94 for the X coordinate of the CoP by the VFA, 0.76 for the Y coordinate of the CoP by the VFA, 0.89 for the X coordinate of the CoP by the WMA, and 0.64 for the Y coordinate of the CoP by the WMA.

**Figure 8:**
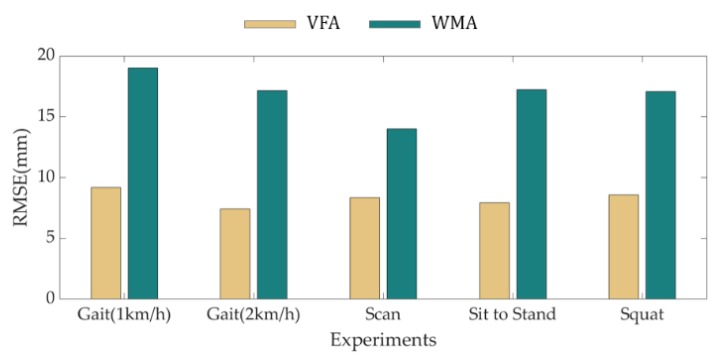
Bar graph of the average RMSE during experiments of all subjects by the VFA (Yellow bar) and the WMA (Green bar).

Figure 8 shows the RMSE of the VFA and the WMA by the reference data of all the subjects during each experiment. The green bars of the WMA have higher values than the yellow bars of the VFA, so it is clear that the error decreased with the proposed algorithm.

## 5. Discussion

In this study, we proposed and validated an algorithm (VFA) for estimating GRF and calibrating CoP of an insole with several experiments. Using eight healthy males our validation experiments confirmed that the VFA can estimate the magnitude of the GRF of an insole during static and dynamic situations with a 29.34 N mean RMSE value and the location of the CoP with an 8.29 mm mean RMSE value. The results also confirmed that the VFA have high correlation coefficients like 0.94 for GRF, 0.94 for the X coordinate, and 0.76 for the Y coordinate. The correlation coefficient of 0.76 can be regarded as a low value but that is because of the low span of the Y coordinate, and it is not low compared with relevant studies. Hu et al. proposed a nonlinear model for estimation of the foot plantar CoP trajectories, and their research had the best success with estimating CoP so far [16]. They achieved an average RMSE value of 2.48 mm for the medial‒lateral direction, which equals the Y coordinate of this study, and 10.18 mm for the anterior‒posterior direction, which equals the X coordinate of this study. The exact distance error was 10.48 mm, which is higher than the 8.29 mm of this study. The experiments done in that study were composed of static experiments like quiet standing, sitting down, and standing up. Those static experiments had input signals of almost all the sensors with monotonous changes of magnitudes of the sensor signals. Those conditions are advantageous for algorithm performance. Moreover, the VFA described in this paper had an average RMSE of 8.28 mm, which is lower even with fewer sensors (six FSR sensors) than the study of Hu et al. (12 FSR sensors). For dynamic situations like walking, we achieved 9.45 mm for the average RMSE value during 1 km/h and 2 km/h gait experiments. Thus, VFA performed the best for estimating the CoP during all situations so far.

Studying the GRF, Park et al. found the NRMSE of estimating the GRF during walking, running, and jumping [13], with values of 14.68 for walking, 10.9 for running and 14.72 for jumping. During the walking, the maximum magnitude of GRF was 800 N. Then RMSE was calculated as 120 N, which was much higher than the error of this study (34.01 N). They had the biggest error during the experiment in 20 to 70% of the gait cycle, while in this study we can see that there is no loss of force during the gait cycle in 0.4 to 0.88 s in Figure 7b. These results confirm that for estimating the GRF, VFA is the best algorithm reported.

**Figure 9:**
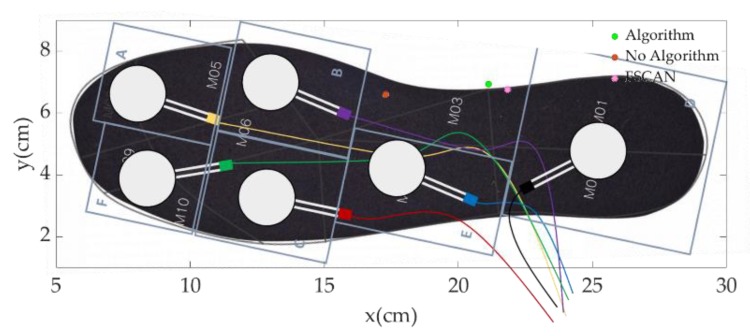
One chosen frame of CoP trajectory plot during the scan experiment by WMA (No Algorithm) with orange point, the VFA (Algorithm) with green point, and the reference data (F-Scan) with pink point.

We concluded that the reason why our proposed algorithm (VFA) has a higher performance than any other algorithms is in virtual forces and moving coordinates. In Figure 6a, the gray circles with bolded black edges indicate the locations of the FSR sensors. With the limitation of the WMA model that has fixed coordinates with weighted constants, the CoP cannot get out of the region of the FSR sensor area, which has the FSR sensors as apexes. We can see that CoPs of WMA are located at the edge which is drawn by connecting the FSR sensors. However, with virtual coordinates, the CoP can move out of the FSR sensor area, as in Figure 6a, with green circles. However, with virtual forces and moving coordinates, CoP can get out of the FSR sensor area, as we can explain through Figure 9.

In Figure 9, we can see three points of CoP by VFA (Algorithm), WMA (No Algorithm) and reference data (F-Scan) of a certain frame during a scan experiment. Of course, the point of the VFA is closer to the F-Scan point than that of WMA. This is because the virtual force of D area which is getting bigger by VFA, drags the orange point of WMA backward. In Table 3, the proportional rate of ADC values of FSR sensors in the A, B, D area is 1:2.4:2.8. However, the proportional rate of the estimated virtual forces of areas is 1:4.45:12.24. The proportion of the force of D area is much bigger than before, so its effect is also higher. The estimated moving X coordinates of C and D areas are also dragged slightly backward compared to before. In the WMA for the calculation of CoP, like $\sum_{i}\frac{F_{i}}{F_{SUM}}x_{i}$, the weighted constant of $x_{i}$ is $\frac{F_{i}}{F_{SUM}}$, so when $F_{i}$ gets bigger, the effect of $x_{i}$ is higher. So, bigger values of virtual forces resulted from the VFA let the coordinates which is slightly dragged backward have more power to drag the CoP to the actual location.

The principle of estimating the virtual forces from other FSR sensors can be explained by linear multiple regression which is regarded as an ANN with one neuron. We fitted the linear multiple regression which has six FSR sensor values for inputs and estimated force of B area for output, as in Equation (3):

(3) $$F_{ER}=a_{0}+a_{1}F_{T}+a_{2}F_{R}+a_{3}F_{L}+a_{4}F_{H}+a_{5}F_{C}+a_{6}F_{LT}$$

We can get the constants for Equation (3) as in Table 4. We know that the constants of A, B, C areas are positive values and those of D, E, F are negative. A positive value means that if the FSR sensor of that constant gets a higher value, it gives the output, which is the estimated force, a positive effect to get higher force. A negative value means the opposite. For example, the constant $a_{4}$ of heel FSR sensor has a negative value, so if the heel FSR sensor gets a higher value, the estimated force of B area gets a lower value. The reason is clear: when the heel gets a higher value, that means the CoP is located in the back side of the foot and FSR sensors on the front side cannot help getting a lower value for pressure. This explains why the protruding areas can be chosen for the locations of the FSR sensors for input values of algorithms.

To sum up, the VFA lets the virtual forces get higher values of pressure than those measured by FSR sensors and also gives the virtual forces more reasonable coordinates than the fixed coordinates of the FSR sensors. So, the CoP that is calculated by a WMA with those virtual forces and moving coordinates can be closer to the actual value. Thus, we can overcome the fundamental limitations of the FSR sensor and use a low-cost sensor like the whole insole measurement pressure sensing system.

The VFA is very practical for the low-cost sensor field. We can use it for the wearable sensor market for weight measurement and daily step analysis with connections to smartphones or computers. Furthermore, in research, as we explained in the introduction, it can be used for rehabilitation engineering, medical engineering, biomechanics and so on. However, in this study, we set a restriction on the size of the foot, but we think it likely that there is an average foot shape for every person and we can make algorithms with the VFA that operate for every foot size. Our future work will be to normalize the algorithms of the virtual forces and coordinates for every size of foot and person to compose only one algorithm for all human beings.

## 6. Conclusions

The CoP and GRF of an insole are the most important parameters for robot and postural control. This study proposed a low-cost flexible insole pressure measurement sensor with FSR sensors that can estimate the foot plantar pressure and its location with very high accuracy compared to existing studies and the WMA. We hope that this algorithm will replace the commercial pressure measurement sensors that have very high prices for the engineering research of biomechanics, biomedical and rehabilitation engineering for fall prevention for the elderly, rehabilitation exoskeleton control, gait analysis and so on.

## Figures and Tables

**Table 1:** Body state information of subjects.

Subject No.	Gender	Age	Height (cm)	Weight (kg)	Shoe Size
1	Male	26	179	67	US8
2	Male	27	180	74	US8
3	Male	24	173	71	US8
4	Male	24	185	77	US8
5	Male	32	173	72	US8
6	Male	34	171	70	US8
7	Male	38	173	79	US8
8	Male	35	169	74	US8

**Table 2:** The RMSE (Root Mean Square Error) of the estimated GRF and the calibrated CoP by VFA and correlation coefficient (CC) of the estimated GRF and the calibrated CoP by VFA and WMA.

Subject No.	RMSE of GRF(N) by VFA					RMSE of CoP(mm) by VFA				
	Scan	Gait 1	Gait 2	Squat	STS	Scan	Gait 1	Gait 2	Squat	STS
1	21.43	26.46	26.79	25.08	19.00	7.43	8.47	6.14	10.56	7.44
2	23.99	35.55	46.28	32.17	16.97	5.38	5.79	8.71	7.36	9.56
3	27.65	45.70	28.11	29.51	17.39	9.94	13.65	10.28	4.38	0.80
4	28.83	34.28	40.35	44.60	38.30	9.74	11.31	4.64	5.75	5.60
5	27.70	23.57	28.41	40.48	20.69	8.38	8.70	7.05	15.5	10.29
6	33.75	29.74	32.98	32.88	15.66	9.40	15.79	8.37	11.90	14.71
7	32.45	54.58	30.80	32.58	20.56	9.49	4.87	7.23	8.58	7.00
8	32.86	22.19	18.04	20.92	14.37	6.98	4.75	7.06	4.61	8.06
Avg.	28.58	34.01	31.47	32.28	20.37	8.34	9.17	7.44	8.58	7.93
	**RMSE of CoP(mm) by WMA**					**CC of GRF by VFA**				
1	15.07	14.27	14.15	14.70	15.40	0.93	0.96	0.95	0.93	0.93
2	11.34	23.07	22.20	18.77	14.19	0.94	0.96	0.98	0.93	0.95
3	17.40	23.06	16.71	16.53	20.60	0.95	0.94	0.97	0.97	0.96
4	12.75	20.86	21.80	12.78	10.9	0.94	0.96	0.94	0.86	0.94
5	11.03	12.05	12.00	17.4	19.44	0.94	0.99	0.99	0.72	0.96
6	18.88	21.11	14.26	20.68	20.10	0.84	0.94	0.96	0.90	0.96
7	10.88	11.49	17.57	14.25	18.05	0.92	0.89	0.97	0.94	0.95
8	14.69	26.17	18.54	21.43	19.12	0.94	0.99	0.99	0.97	0.95
Avg.	14.01	19.01	17.15	17.07	17.23	0.93	0.95	0.97	0.90	0.95
	**CC of CoP X by VFA**					**CC of CoP Y by VFA**				
1	0.99	0.99	0.99	0.83	0.74	0.96	0.88	0.70	0.62	0.46
2	0.99	0.99	0.99	0.96	0.96	1.00	0.71	0.60	0.65	0.77
3	0.98	0.98	0.98	0.73	0.77	0.97	0.97	0.94	0.66	0.62
4	0.99	0.96	1.00	0.84	0.64	0.99	0.84	0.70	0.46	0.26
5	1.00	0.99	1.00	0.91	0.94	0.98	0.92	0.87	0.68	0.43
6	0.98	0.98	0.99	0.93	0.98	0.96	0.69	0.54	0.55	0.65
7	0.99	0.98	0.98	0.85	0.88	0.96	0.86	0.75	0.69	0.71
8	0.99	0.99	0.99	0.97	0.98	0.97	0.84	0.67	0.86	0.90
Avg.	0.99	0.98	0.99	0.88	0.86	0.97	0.84	0.72	0.65	0.6
	**CC of CoP X by WMA**					**CC of CoP Y by WMA**				
1	0.98	0.97	0.98	0.89	0.65	0.94	0.65	0.40	0.06	0.33
2	0.96	0.95	0.94	0.92	0.94	0.99	0.67	0.35	0.58	0.69
3	0.97	0.96	0.98	0.59	0.68	0.96	0.91	0.75	0.60	0.62
4	0.98	0.93	0.96	0.70	0.18	0.98	0.61	0.85	0.42	0.13
5	0.99	0.97	0.98	0.92	0.92	0.82	0.69	0.71	0.72	0.31
6	0.95	0.94	0.97	0.74	0.96	0.93	0.50	0.49	0.51	0.58
7	0.98	0.96	0.96	0.85	0.89	0.95	0.80	0.55	0.52	0.55
8	0.98	0.97	0.95	0.95	0.95	0.93	0.78	0.31	0.78	0.86
Avg.	0.97	0.96	0.97	0.82	0.77	0.94	0.70	0.55	0.52	0.51

Gait 1 means gait at 1 km/h, Gait 2 means walking at 2 km/h and STS means sit to stand.

**Table 3:** Numerical values of frame in Figure 9.

Numerical Value	Divided Area of Insole					
	A	B	C	D	E	F
**ADC Value**	55	132	0	154	0	0
**Estimated Virtual Force (N)**	23.418	104.242	0	286.743	0	0
**Proportional Rate of ADC Value**	1	2.4	0	2.8	0	0
**Proportional Rate of Estimated Virtual Force**	1	4.45	0	12.24	0	0
**X Coordinate of FSR Sensor**	7	12	12	25.5	9.25	3.5
**Estimated Moving X Coordinate of Divided Area**	5.684	12.034	*NaN*	25.692	*NaN*	*NaN*

*NaN* means “Not a Number” because we didn’t estimate the coordinates of areas if there were no pressure value on the FSR sensors.

**Table 4:** Fitted constants for multiple linear regression of estimated force of B area.

	Constants						
	$a_{0}$	$a_{1}$	$a_{2}$	$a_{3}$	$a_{4}$	$a_{5}$	$a_{6}$
**Value**	79.160	0.316	3.155	0.321	−0.387	−0.016	−1.253
